# From Rhizosphere to Resistance: Microbe‐Plant Interactions in Eco‐Smart Biocontrol

**DOI:** 10.1002/mbo3.70398

**Published:** 2026-09-28

**Authors:** Shilpy Singh, Varun Kumar Sharma, Dharmsheel Shrivastav, Jayant M. Kushwaha, Manoj Kumar Mishra, Mirza Masroor Ali Beg

**Affiliations:** ^1^ Department of Biotechnology and Microbiology, School of Sciences Noida International University Greater Noida Uttar Pradesh India; ^2^ Department of Biotechnology SR Institute of Management & Technology Lucknow Uttar Pradesh India; ^3^ Faculty of Medicine Ala‐Too International University Bishkek Kyrgyzstan

**Keywords:** artificial intelligence in agriculture, eco‐smart biocontrol, induced systemic resistance, plant–microbe interactions, rhizosphere microbiome

## Abstract

The increasing limitations of chemical pesticides such as environmental pollution, pathogen resistance, and threats to human and ecosystem health have increased the demand for sustainable, biologically based crop protection methods. Eco‐smart biocontrol has emerged as a game‐changing paradigm that uses beneficial microorganisms associated with plants to suppress phytopathogens, boost plant immunity, and make agroecosystems more resilient over time. Moving beyond traditional single‐strain biocontrol, eco‐smart biocontrol integrates multi‐omics discovery, artificial intelligence‐assisted predictive microbiome design, and dynamic rhizosphere ecology. This review brings together ecological, molecular, and technological dimensions of eco‐smart biocontrol, focusing on the rhizosphere as a dynamic hotspot for plant–microbe interactions. We investigate rhizosphere microbiome assembly and demonstrate the preferential recruitment of beneficial bacteria, fungi, actinomycetes, and mycorrhizal symbionts by plant root exudates. Moreover, the review highlights the impact of innovations in multi‐omics techniques (metagenomics, transcriptomics, proteomics, and metabolomics), systems biology, and artificial intelligence on microbial biocontrol agent discovery, functional validation, and predictive design. Examples from cereal crops, legumes, and horticulture crops indicate that the application of beneficial microbial inoculants can significantly lower the burden of pests and diseases, enhance crop productivity, and fit perfectly within an integrated pest management system. Lastly, we critically analyze the main challenges preventing large‐scale adoption, such as inconsistent field performance, limited microbial survival and competitiveness, and comparative regulatory frameworks across global markets. Ultimately, eco‐smart microbial biocontrol combines mechanistic insights with omics‐driven discovery, artificial intelligence (AI)‐ assisted prediction, advanced formulation strategies, and field‐level validation, creating a strong, scalable, and environmentally friendly framework for resilient, low‐input agricultural systems.

## Graphical abstract

Conceptual framework of eco‐smart biocontrol: Root exudates released by plants shape rhizosphere microbial community assembly by selectively recruiting beneficial microorganisms. These microbes suppress phytopathogens through direct mechanisms such as antibiosis, parasitism, enzyme production, and niche competition, while simultaneously activating indirect plant defenses via induced systemic resistance (ISR) and systemic acquired resistance (SAR). The integration of pathogen suppression and microbe‐mediated immune priming enhances plant health, stress tolerance, and productivity. Collectively, these processes reduce dependence on chemical pesticides and promote improved yield, crop quality, and long‐term agroecosystem sustainability.

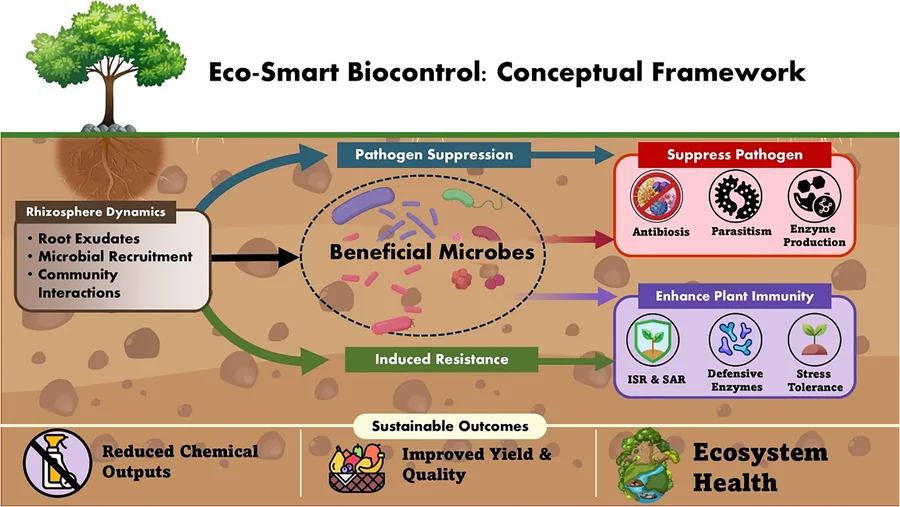

## 1 Introduction

The current trend in farming is to produce high yields without causing any harm to the environment or human beings due to the increased use of pesticides. Even though the application of pesticides has helped reduce plant diseases and yield high amounts of crops, continued dependency on such chemicals has caused pesticide resistance, loss of biodiversity in soils, food contamination, and disrupted ecosystem services (Carvalho 2017; Nicolopoulou‐Stamati et al. 2016; Hua and Liu 2024). These restrictions, along with stricter rules and regulations and a growing demand from consumers for food that doesn't leave behind any residue, have sped up the shift toward plant protection methods that are more environmentally friendly and based on biology.

In this scenario, eco‐smart biocontrol has emerged as a revolutionary concept combining ecology, microbial action, and systemic thinking for sustainable management of plant diseases. Unlike traditional biocontrol, which relies on the empirical, singular application of an isolated antagonistic strain that frequently suffers from unpredictable field survival, eco‐smart biocontrol is a systemic, knowledge‐intensive paradigm. It leverages microbial diversity, functional redundancy, multi‐strain consortia, and compatibility with the plant genotype and surrounding agroecosystem (Mishra et al. 2025; Keswani et al. 2020; Compant et al. 2021). This framework shifts the intervention strategy from treating symptoms with a single “biological fungicide” to engineering a resilient, self‐sustaining rhizosphere microbiome that continuously communicates with the plant host via root exudates, small RNAs, and induced systemic resistance. This framework closely mirrors the goals of climate‐smart agriculture, sustainable intensification, and the One Health concept, ensuring crop protection safeguards environmental and human health while minimizing ecological disruption (Compant et al. 2024; Latini 2025).

The core of eco‐biocontrol technology revolves around the rhizosphere microbiome, which is characterized by constant interactions between plant roots, physical and chemical properties of soil, and various microorganisms. The plant roots release chemicals that act as selection factors for beneficial microbes and help regulate microbial populations in the rhizosphere microbiome (Vives‐Peris et al. 2020; Wankhade et al. 2025). On the other hand, the microbes residing within the rhizosphere provide nutrients, counteract pathogens, and reduce stress to the plants (Bhadrecha et al. 2023; Fitzpatrick et al. 2020).

Rhizosphere‐beneficial bacteria and fungi, such as *Bacillus, Pseudomonas, Trichoderma*, arbuscular mycorrhizal fungi, and endophyte communities, are crucial players in nutrient turnover, phytohormone synthesis, and boosting plant immune functions (Höll and Bossert 2022; Harman et al. 2021). Such microbes inhibit pathogens via several synergistic methods, including antibiosis, nutrient and niche competition, mycoparasitism, disruption of quorum sensing, and manipulation of microbial consortia (Köhl et al. 2019; Yang et al. 2024). Critically, several beneficial microorganisms induce systemic acquired resistance (SAR) and priming of defense mechanisms, which enable the plant to respond quickly and effectively to subsequent pathogenic assaults (Yu et al. 2022; Xiao et al. 2025).

The drawbacks of chemical pesticides only serve to emphasize the need to develop microbial pesticides. Pesticides have led to the development of pest resistance, poor effectiveness, and costly effects on the farmers (Corkley et al. 2022; Hawkins et al. 2019). Pesticide residues also negatively impact soil microbiota that play a critical role in soil fertility and plant growth (Thakur and Sharma 2024; Maharana et al. 2024). The benefits of microbial pesticides include their biodegradable nature, environmental friendliness, and ability to operate effectively alongside other microbial communities, among others.

While there have been some promising developments in this direction, the widespread application of microorganisms as a biological control measure continues to be hindered by the unpredictable performance in the field, poor stability, variability in host reaction, and limited knowledge on the mechanism involved in plant–microbe interaction within the highly dynamic environment (Sokra et al. 2025; Sharma et al. 2023). Some of the notable scientific advancements made through next‐generation omics, systems biology, and computational analysis include metagenomics, metabolomics, transcriptomics, and artificial intelligence‐based approaches (Singh et al. 2024a; Kumar et al. 2024; Guo et al. 2024; Bai et al. 2022). These tools are changing the way biocontrol is designed by making it possible to find keystone taxa, functional traits, and predictive markers of microbial efficacy.

This review provides an overview of the recent progress made in developing eco‐friendly biocontrol strategies, especially those based on the interactions between plants and microbes that occur in the rhizosphere environment and lead to the induction of plant resistance to diseases. The study also emphasizes understanding the underlying ecological, physiological, and molecular principles of microbial biocontrol. In addition, the paper discusses formulation strategies, field performance, and current barriers that prevent the application of biocontrol strategies in an economically viable manner. In summary, this literature review attempts to develop a comprehensive approach towards formulating eco‐friendly biocontrol measures through the integration of basic research and its application in agriculture. The general concept of eco‐smart biocontrol strategy, which incorporates rhizosphere microbiota recruitment, pathogen suppression, and induction of the plant defense system.

## 2 The Rhizosphere as a Dynamic Microbial Hotspot

The spatial structure and functionality of the rhizosphere, comprising specific compartments and interaction zones within it, are well‐represented in Figure 1. Rhizosphere is defined as an ecologically distinct part of soil, heavily affected by plant roots, and is known for its extreme physico‐chemical gradients and biological activities. This microhabitat can be described using rhizodeposition, consisting of the steady secretion of root exudates, mucilage, border cells, and shed plant tissues (Wankhade et al. 2025; Yue et al. 2023). As a result of this, microbial communities found in the rhizosphere are different from those found in bulk soil in terms of taxonomy, metabolism, and function. The rhizosphere is positioned as a key hub for biological disease control, nutrient exchange, and plant–microbe communication thanks to these root‐driven activities.

**Figure 1:**
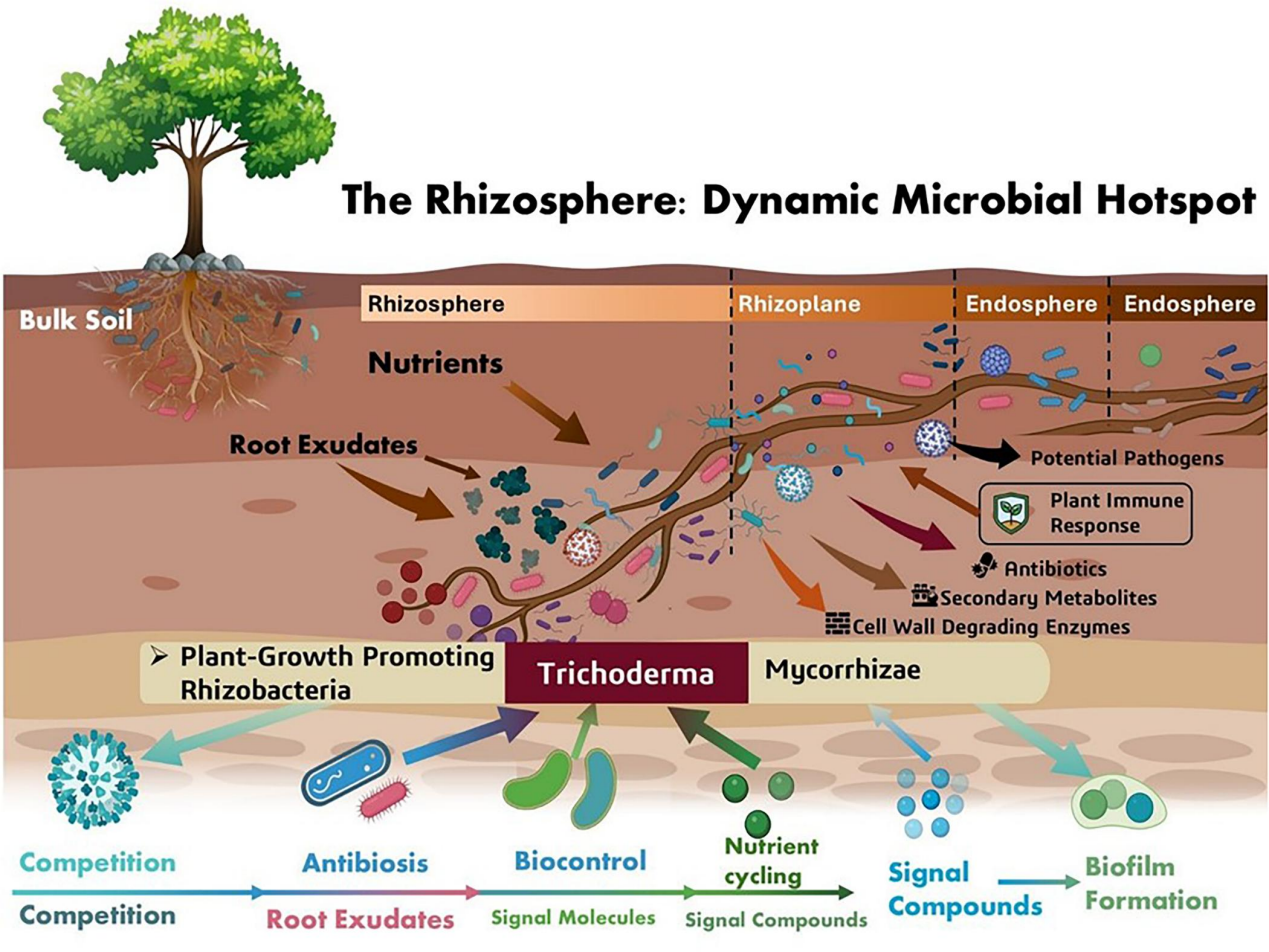
The rhizosphere as a dynamic microbial hotspot. Schematic representation of the rhizosphere as a highly active soil–root interface comprising distinct compartments, including bulk soil, rhizosphere, rhizoplane, and endosphere. Root exudates released by plant roots create nutrient and signaling gradients that selectively recruit and enrich diverse beneficial microorganisms such as plant growth‐promoting rhizobacteria, *Trichoderma*, and mycorrhizal fungi. These microbes interact through competition, antibiosis, biofilm formation, nutrient cycling, and signal exchange, collectively shaping microbial community structure and function. The coordinated activity of these microbial assemblages suppresses potential pathogens, modulates plant immune responses, and supports plant growth, health, and resilience under dynamic environmental conditions.

### 2.1 Composition of Rhizosphere Microbiota: Bacteria, Fungi, Actinomycetes, Archaea, and Protists

The rhizosphere microbiome is characterized by an extremely diverse community consisting of bacteria, fungi, actinomycetes, archaea, and protists, which play different roles for plant well‐being and soil ecology. Bacteria make up the most significant proportion of microbes and can swiftly adjust to root‐produced organic compounds, which leads to high numbers of Proteobacteria, Actinobacteriota, Bacteroidota, and Firmicutes in soils surrounding roots in agricultural production (Ahsan et al. 2024; Xiong et al. 2024). Most of these groups can serve as plant growth‐promoting rhizobacteria (PGPR).

The importance of fungi such as saprophytes, endophytes, and arbuscular mycorrhizal fungi (AMF) cannot be understated in terms of breaking down organic material, acquiring nutrients, and building soil aggregates, all while developing extensive interaction systems with bacterial populations (Duan et al. 2025; Lavado and Chiocchio 2025). Actinomycetes, especially species of Streptomyces, are distinguished by their filamentous nature and abundant synthesis of antibiotic substances, thus constituting important components of naturally disease‐suppressive soils (Khan et al. 2023).

Though their numbers may be relatively low, archaea are involved in important metabolic processes, such as nitrification and methane metabolism, especially in cases of nutrient limitation or high‐stress environments (Kumar et al. 2025; Praveen and Singh 2024). Protists like amoebae and flagellates function as microbial predators by controlling bacterial growth patterns, recycling nutrients, and indirectly promoting beneficial plant properties in bacteria (Taerum et al. 2025; Wang et al. 2024). These interacting microbial groups work together to make a tightly connected, multitrophic network that helps nutrients move around, keeps pathogens from growing, and makes plants stronger in the rhizosphere.

### 2.2 Root Exudates as Microbial Modulators: Sugars, Amino Acids, Phenolics, and Flavonoids

The composition of root exudates is made up of complex chemicals of various molecular weights, including simple sugars, amino acids, organic acids, phenols, flavonoids, and special secondary metabolites, which act both as sources of nutrients and chemical messengers (Wankhade et al. 2025; Yue et al. 2023). The simple sugars like glucose, fructose, and sucrose are quickly consumed by the rhizosphere bacteria and thus influence chemotaxis and colonization of the roots by the bacteria (Lei et al. 2023; Zhao et al. 2023).

On the other hand, amino acids provide not only carbon and nitrogen but also play a role in the regulation of gene expression as well as competitive abilities of microbes in the rhizospheric niche. However, secondary metabolites like phenolics and flavonoids have more impact on the selection process within microbial communities. For instance, particular phenolic compounds inhibit the activity of soil‐born pathogenic microorganisms and stimulate the proliferation of beneficial microbes, including PGPR and nitrogen fixers (Sibanyoni et al. 2025). Meanwhile, flavonoids are involved in mutualistic plant–microbe relationships as symbiotic signal inducers (Wang et al. 2022; Zhang et al. 2022).

Notably, the components and amounts of root exudates are extremely plastic, being determined by the genetic makeup of the plant, its development stage, nutrient requirements, and the presence of any stress factors from the environment. This plasticity allows plants to modify the community of microorganisms that form around them according to their physiological needs and environmental conditions (Yue et al. 2023; Lei et al. 2023).

### 2.3 Microbial Community Assembly and Succession in the Rhizosphere

Rhizosphere microbial community establishment involves selective and stochastic processes dependent on the interaction between characteristics of the host plant, the soil, and the interactions in the microbial community. Roots serve as ecological filters, allowing only microorganisms that can metabolize root exudates and withstand the specific conditions of the rhizosphere, including variation in oxygen levels, pH, and nutrient concentrations (Yue et al. 2023; Pantigoso et al. 2022).

Host genetics and development play significant roles in determining the composition of rhizosphere microbiomes, where unique microbiota communities develop depending on plant growth stages based on the physiological demands of each phase (Yue et al. 2023; Hesen et al. 2023). After the first wave of microbes has been recruited to the rhizosphere, microbial succession ensues as pioneer microbes alter their environment by reducing nutrients, producing metabolites, and creating niches, affecting the subsequent arrival of other microorganisms. This pattern is similar to classic ecological succession, resulting in changes in the dominant species.

Moreover, microbial interrelationships, which involve competition, mutualism, cross‐feeding, and grazing by protozoa, further aid in fine‐tuning community architecture and functional stability. These relationships play an important role in the formation of robust microbial consortia that help in stabilizing rhizosphere functions necessary for plants (Bai et al. 2022; Yue et al. 2023).

## 3 Mechanisms of Microbial Biocontrol and Competitive Dynamics

Microbial biocontrol involves the combined application of ecological and molecular strategies that lead to effective control of phytopathogens, regulation of host defense responses, and rhizosphere stability. Rather than operating independently, plant–microbe interactions and competitive mechanisms act cooperatively within microbial consortia and plant–soil environments to improve persistence and reliability in disease suppression (Trivedi et al. 2020; Maciag et al. 2023; Carezzano et al. 2023). The primary biocontrol approaches encompass nutrient competition, direct antibiosis, mycoparasitism, quorum‐sensing interference, and robust root colonization.

### 3.1 Competition for Nutrients and Ecological Niches

Strong competition exists among microorganisms for organic carbon substrates produced by plant roots, micronutrients like iron, and spatial colonization niches present in the root environment. Siderophore synthesis serves as a critical competitive mechanism because it enables beneficial microorganisms such as *Pseudomonas fluorescens* and *Bacillus subtilis* to effectively sequester ferric iron in the soil matrix, restricting access by competing phytopathogens and suppressing their virulence (Shao et al. 2025; Sánchez et al. 2026; Verma et al. 2023; Singh et al. 2022). Furthermore, spatial niche competition through rapid root colonization and biofilm formation prevents plant pathogens from occupying infection courts on the root epidermis.

### 3.2 Direct Antagonism, Antibiosis, and Volatile Organic Compounds (VOCs)

Antagonistic competition is characterized by the direct suppression of competing microorganisms through the production of antimicrobial secondary metabolites, lytic enzymes, volatile organic compounds, and quorum‐sensing inhibitors (Sardans et al. 2023; Chlebek et al. 2022). Plant growth‐promoting microorganisms and fungi produce a diverse array of antimicrobials, including cyclic lipopeptides (*surfactin*, *iturin*, and *fengycin* derived from *Bacillus* species) that disrupt pathogen cell membranes, causing rapid cell leakage and lysis (Pedrozo and Nicolli 2026; Saiyam et al. 2024). Additionally, *Pseudomonas* and *Streptomyces* strains synthesize specialized aromatic antibiotics such as phenazines, pyrrolnitrin, pyoluteorin, and 2,4‐diacetylphloroglucinol (2,4‐DAPG) that interfere with cellular metabolism and oxidative reactions (Wu et al. 2026; Gómez‐Pérez et al. 2024). Volatile organic compounds like hydrogen cyanide, acetoin, and 3‐hydroxy‐2‐butanone travel through the soil matrix to limit pathogen growth while simultaneously priming host plant defense genes.

### 3.3 Parasitism and Predation of Pathogens

Direct parasitism and predation constitute highly efficient, taxon‐specific mechanisms of biological control. This approach is characteristically exhibited by fungal mycoparasites such as *Trichoderma* and *Gliocladium* species, which recognize the hyphae of soil‐borne fungal pathogens, coil around them, and secrete cell wall‐degrading enzymes including chitinases, β−1,3‐glucanases, and proteases (Woo et al. 2022; Dou et al. 2022). These enzymes degrade pathogen cell walls, resulting in hyphal lysis and providing nutrients for the mycoparasite while concurrently releasing microbe‐associated molecular patterns (MAMPs) that stimulate plant defense responses (Mikiciuk et al. 2024). Similarly, predatory bacteria like *Lysobacter* spp. and *Bdellovibrio bacteriovorus* prey on and lyse pathogenic bacteria, regulating microbial population dynamics within the rhizosphere (Martins et al. 2022).

### 3.4 Quorum‐Sensing Interference and Signal Disruption

Many plant pathogens rely on quorum‐sensing (QS) systems to coordinate the production of virulence factors, biofilm formation, and host infection. Quorum quenching through enzymatic interference offers an eco‐friendly biocontrol strategy (Wu et al. 2026; Zhu et al. 2023). Beneficial rhizosphere microorganisms secrete specific enzymes, such as lactonases, acylases, and oxidoreductases, which degrade or modify primary QS signaling molecules like *N*‐acyl‐homoserine lactones (AHLs). By inhibiting the accumulation of these signals, quorum quenching disrupts concerted pathogen virulence without exerting strong selection pressure toward resistance development (Zhu et al. 2023).

### 3.5 Biofilm Formation and Root Colonization

Sustained biocontrol efficacy requires efficient and stable root colonization. Bacterial attachment and extracellular polymeric substance (EPS) matrix production enable beneficial taxa, including *Bacillus*, *Pseudomonas*, and endophytic strains to form structured biofilms on root surfaces (Kumar and Singh 2020; Kalam et al. 2017; Riseh et al. 2025). These biofilms create a physical barrier against pathogen invasion, maintain prolonged association with the host plant to continuously trigger induced systemic resistance (ISR), and offer enhanced resilience against environmental abiotic stresses. These multifaceted interactions and defense pathways are comprehensively illustrated in Figure 2.

**Figure 2:**
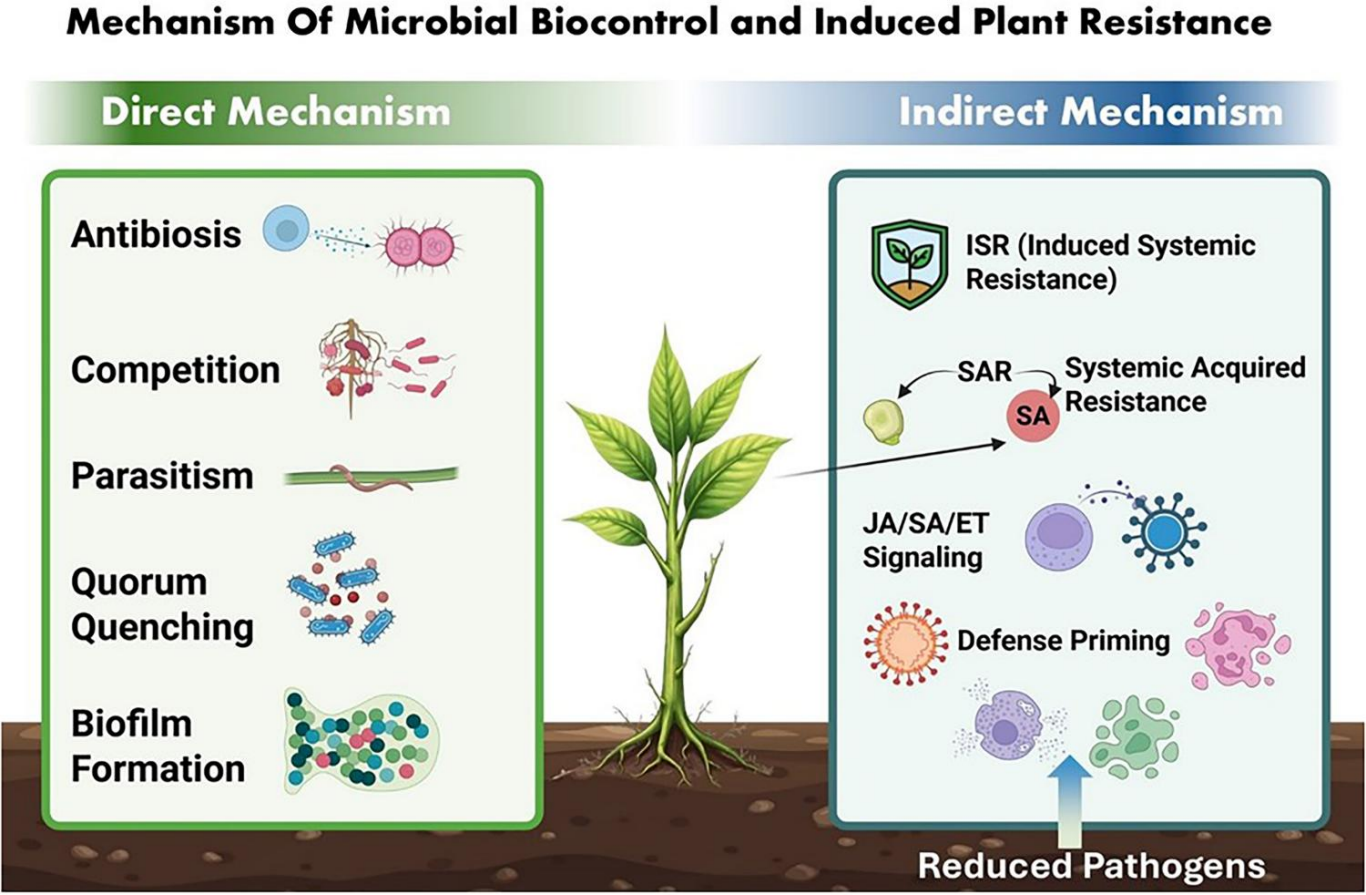
Mechanisms of microbial biocontrol and induced plant resistance. Schematic illustration of the direct and indirect mechanisms by which beneficial microorganisms suppress plant pathogens and enhance host immunity. Direct mechanisms include antibiosis, competition for nutrients and niches, parasitism, quorum‐sensing interference, and biofilm formation, which collectively reduce pathogen survival and establishment in the rhizosphere. Indirect mechanisms involve activation of plant defense pathways through induced systemic resistance (ISR) and systemic acquired resistance (SAR), mediated by jasmonic acid (JA), salicylic acid (SA), and ethylene (ET) signaling, as well as defense priming. The integration of these complementary strategies results in reduced pathogen pressure, enhanced plant resilience, and improved crop health under eco‐smart biocontrol systems.

## 4 Induced Plant Resistance Mediated by Microbes

Beyond the suppression of pathogens, the role of beneficial microbes involves inducing immune responses in the plant, thereby improving the ability of the plant to defend itself against any pathogen attacks. The induction of immune responses in the plant is carried out via specific signaling networks, transcriptional reprogramming, and immune memory, rather than through constant activation of the plant's immune system. ISR and SAR, along with other related concepts such as hormone regulation and defense priming, are some of the important aspects of sustainable agricultural practices (Xiao et al. 2025; Pieterse et al. 2014; Hönig et al. 2023).

### 4.1 Induced Systemic Resistance (ISR)

ISR, on the other hand, is a condition where plants exhibit increased immunity due to nonpathogenic rhizosphere and endosphere microbes that do not cause any infections while colonizing the roots. Induced systemic resistance provides non‐host specificity and is mainly governed by jasmonic acid (JA) and ethylene (ET) hormone signaling pathways rather than salicylic acid (SA)‐dependent responses (Xiao et al. 2025; Pieterse et al. 2014).

*Pseudomonas fluorescens, Bacillus subtilis*, and arbuscular mycorrhizal fungi have been well‐documented as effective inducers of ISR. Such microorganisms secrete MAMPs, siderophores, and VOCs, which are recognized by plant receptors, leading to systemic immune priming (Hönig et al. 2023; Srikamwang et al. 2023). In contrast to the activation of defense responses by plant cells, ISR is associated with the generation of a primed physiological condition in plants, characterized by increased responsiveness to signals from pathogens, triggering more rapid and efficient defense mechanisms.

ISR is especially beneficial for eco‐friendly biocontrol since it provides immunity to a variety of pests, ranging from fungal to bacterial and insect species, all while incurring minimal physiological costs on the plant during normal development (Xiao et al. 2025; Bai et al. 2022).

### 4.2 Systemic Acquired Resistance (SAR) and Crosstalk with ISR

SAR, or Systemic Acquired Resistance, refers to a sustained immune reaction that usually follows a localized infection by a pathogen, resulting in resistance throughout the organism. SAR is often linked to the biosynthesis of SA and PR proteins (Zholdasbek et al. 2026; Spoel and Dong 2024), which offer adequate protection against biotrophic and hemibiotrophic pathogens.

Even though ISR and SAR had traditionally been considered as two separate mechanisms, accumulating data suggest that there is substantial cross‐talk between these defense mechanisms. Beneficial bacteria may affect SAR either through modulation of SA synthesis and signal transduction or indirectly via priming of SA‐induced gene expression without inducing expensive defense responses (Hönig et al. 2023; Hou and Tsuda 2022). It has been observed that ISR‐inducing rhizobacteria induce the sensitivity of SAR‐related genes allowing for immediate activation of defense in response to pathogen attack.

The interaction between hormone and gene regulation permits the plant to finely adjust its immunity response based on the nature of the pathogen and its environment, switching between the JA/ET‐controlled ISR and the SA‐regulated SAR systems whenever necessary.

### 4.3 Hormonal Signaling Networks: Jasmonic Acid, Salicylic Acid, and Ethylene

The role of plant hormones includes being key regulators for microbial signals and immune responses. In particular, jasmonic acid (JA), salicylic acid (SA), and ethylene (ET) are the primary signaling molecules regulating induced resistance by virtue of their cooperative and competitive effects (Li et al. 2022).

The JA signaling pathway plays a critical role in ISR and protection against necrotrophic pathogens and insect pests. Beneficial microorganisms upregulate the synthesis of JA and downstream transcription factors, which results in higher gene expression levels of defense‐related genes like PDF1.2 and production of antimicrobial compounds (Roychowdhury et al. 2024).

SA is the main controller of SAR, acting as an inducer of resistance to biotrophic plant pathogens through the expression of PR proteins (PR1, PR5) and immune responses. Modification of SA pathway by microbes can increase immune response without wasting energy on defense when there are no pathogens (Spoel and Dong 2024; Hou and Tsuda 2022).

ET serves as an important regulator that adjusts JA‐ and SA‐induced responses. The involvement of ET‐related signal transduction pathway components, such as EIN2 and ERFs, is common to activate ISR and contributes to context‐dependent immune responses (Li et al. 2022). Interactions between these hormone pathways allow plants to adapt their immunity in relation to different stimuli.

### 4.4 Defense Priming and Immune Memory

Plant defense priming is another characteristic of microbial resistance that can be described as an economically favorable approach where plants improve their defense ability without activating defense mechanisms continuously. When plants are stimulated by pathogens, they show rapid and enhanced transcription and metabolic responses, such as fast induction of PR genes, WRKY transcription factors, and ROS‐producing enzymes (Hönig et al. 2023).

Rhizobacteria, mycorrhizae, and endophytes, known to be beneficial for plants, can trigger systemic priming and thereby train the immune system of the plant to react efficiently against future biotic challenges. It has been found recently that priming may rely heavily on epigenetics such as histone methylation and acetylation, leading to rapid activation of the genes associated with defense (Qadir et al. 2026).

Sometimes, such epigenetic tags remain stable during development or even from one generation to another, thereby serving as a mechanism for immune memory and disease resistance without causing any significant fitness cost.

Microbe‐induced resistance combines ISR, SAR, hormone‐based signaling, and defense priming in order to develop a multilevel immunity system that helps plants become resilient against different pathogens. By regulating JA, SA, and ET signals and generating immune memory, beneficial microbes help plants achieve a dynamic equilibrium between growth and defense in nature. Such processes play key roles in eco‐intelligent biocontrol, which is considered an environmentally friendly option to synthetic fungicides (Charpe et al. 2025).

## 5 Molecular Dialogues in the Rhizosphere

Rhizospheres are molecular communication interfaces that are very dynamic, as opposed to being simple physical boundaries between roots and soil. Herein, plants and microbes engage in chemical signals, protein, RNA, and metabolite exchanges that control root colonization, immune responses, nutrient cycling, and microbial community dynamics (Ren et al. 2026). The development of transcriptomics, metabolomics, and multi‐omics approaches has demonstrated that these molecular communications constitute signaling networks and ultimately dictate if plant–microbe interactions yield beneficial outcomes through mutualism, primed immunity, or defense responses (Singh et al. 2024a; Li et al. 2022).

### 5.1 Microbial Elicitors and Effectors

Microbial elicitors are conserved or semi‐conserved compounds synthesized by microbes and detected by plants to trigger immune or developmental signals. Microbial elicitors include MAMPs such as flg22, lipopolysaccharide (LPS), peptidoglycan, and chitin oligomers in fungal cell walls (Lü et al. 2022). Detection of microbial elicitors triggers Pattern‐Triggered Immunity (PTI), which is characterized by an increase in calcium, ROS bursts, MAPK activation, and defense gene expression (Deng et al. 2026).

On the other hand, pathogens utilize effector proteins that interfere with the host's immune system by inhibiting or escaping the plant's defense systems by acting on signaling nodes in the host. For instance, effectors affect the receptor‐linked kinases and immune‐related components like BAK1 and BIK1, hence interfering with the amplified signaling pathways and subsequent defenses (Xiang et al. 2025). Interestingly, recent studies reveal that the beneficial microorganisms have effectors or effector‐like molecules that manipulate the host's signaling pathways in a manner that enables them to colonize but do not trigger any immune response (Atanasković et al. 2025).

### 5.2 Plant Pattern Recognition Receptors and Signal Discrimination

Recognition of signals produced by microbes is primarily mediated by pattern recognition receptors (PRRs) in the plasma membrane. PRRs detect conserved features of microbes and trigger PTI through a cascade of signaling pathways, including MAPKs, transcription factors, and defense‐related hormones. One of the best studied examples of such PRRs is the receptor for flagellin, FLS2.

However, the role of PRRs in immunity is not limited to acting simply as binary immune switches. Plant systems have evolved signal differentiation capabilities, allowing them to differentiate pathogens from beneficial microorganisms. As an example, rhizobial symbiotic signaling molecules, like Nod factors, are detected through LysM domain receptor proteins that initiate symbiotic signal cascades while inhibiting the activation of potent immune responses (Jones et al. 2007). The same principle has also been seen in interactions with mycorrhizal fungi and endophytic bacteria (Valente et al. 2025).

### 5.3 Small RNAs and Epigenetic Regulation in Cross‐Kingdom Communication

Smaller RNAs, such as miRNAs and siRNAs, have been identified as essential modulators of plant–microbe interactions in recent years. In the context of plants, small RNAs help regulate immunity and metabolism through gene expression modulation in response to the presence of microbes.

Recent research showed cross‐kingdom RNA trafficking, where small RNAs generated from plants can be released to the rhizosphere and subsequently absorbed by microbes to regulate their behavior and gene expression. In addition, microbes have the capacity to release small RNAs to the plant cells to inhibit the plant's immune system or affect its metabolism (Niño‐Sánchez et al. 2026; Cai et al. 2018; Middleton et al. 2024). The transfer of RNA between plants and microbes is a form of molecular interaction that complements ligand‐receptor interactions.

Epigenetic factors play an important role in integrating microbe signals within the long‐term responses of plants. Histone modifications, chromatin remodeling, and DNA methylation status of certain defense genes could lead to memory responses, where plants learn to respond faster under repetitive stress conditions (Abdulraheem et al. 2024). Epigenetic reprogramming connects a temporary interaction of plants with microbes to a permanent change in their defense capability.

### 5.4 Metabolomics of Plant–Microbe Communication

Metabolomics has shown promise as an innovative tool to understand the chemical communication within the rhizosphere. Root exudates are made up of sugars, amino acids, organic acids, flavonoids, phenolic compounds, and other metabolites, which not only act as nutrients but also signal molecules to recruit, regulate, and influence microbial communities (Gupta et al. 2022).

The use of mass spectrometry‐based technology (LC‐MS, GC‐MS, and imaging metabolomics) facilitates the detection and localization of these metabolites in roots at high resolution. Metabolomics studies have revealed that some profiles of root exudates facilitate the proliferation of symbiotic microorganisms and inhibit pathogenic species, thus establishing disease‐suppressive rhizospheres (Liao et al. 2025).

Metabolomics also encompasses feedback responses of microbes to changes in plant physiology. The impact of microbial secondary metabolites like lipopeptides, volatile organic compounds (VOC), and phytohormone analogs include changes in root architecture and priming of systemic resistance. Metabolomics in conjunction with transcriptomics has elucidated the correlation between metabolite profiles and genes involved in induced systemic resistance (Gayithri et al. 2026).

Molecular conversations between plant roots and microorganisms in the rhizosphere are due to intricate signaling networks that include microbial elicitors and effectors, plant PRR‐triggered perception, regulation by small RNAs, and chemical diversity among metabolites. The integration of these signals will decide the course of interactions between plants and microbes, whether immune responses will be triggered or symbiotic partnerships established. Multi‐omics approaches are providing new insights into these interactions, showing how plants and microorganisms make sense of their molecular world to enhance survival and competitiveness. Unraveling these interactions is key to designing intelligent ecological biocontrol interventions using natural communication systems (Verma et al. 2023).

## 6 Role of Beneficial Microbial Groups in Biocontrol

Positive microbial groups form the operational foundation of biological control methods used in sustainable farming. They minimize the reliance on chemical pesticides by inhibiting pathogens, stimulating plant resistance, and boosting nutrient absorption capabilities (Rezaee Danesh et al. 2025). Some significant microbial groups that play crucial roles in biological control processes are rhizobacteria, fungal biocontrol agents, endophytes, and mycorrhizae, which utilize different but interconnected mechanisms to foster eco‐friendly biological control systems (Verma et al. 2023). The following table shows some of the most common microbial groups involved in eco‐friendly biological control processes along with their predominant antagonistic and immunomodulation activities and induced plant responses towards the pathogen‐crop system (Table 1).

**Table 1:** Major beneficial microbial groups involved in eco‐smart biocontrol, their dominant mechanisms, plant defense outcomes, and target pathosystems.

Microbial group	Representative genera/species	Dominant biocontrol mechanisms	Induced plant responses	Target pathogens/crops	References
Rhizobacteria (PGPR)	*Bacillus subtilis*, *B. velezensis*	Lipopeptides (surfactin, iturin, fengycin), CWDEs, biofilm formation, ISR induction	JA/ET‐mediated ISR, cell wall reinforcement, ROS priming	*Fusarium*, *Rhizoctonia*, nematodes (cereals, vegetables)	Saiyam et al. (2024)
	*Pseudomonas fluorescens*, *P. putida*	Antibiotics (2,4‐DAPG, phenazines), siderophores, VOCs, quorum quenching	ISR, enhanced iron uptake, SA‐JA crosstalk	*Rhizoctonia solani*, *Pythium*, *Erwinia*	Yu et al. (2022)
Actinomycetes	*Streptomyces* spp.	Antibiotics, VOCs, CWDEs, niche exclusion	Defense gene activation, growth promotion	Soilborne fungi and bacteria (legumes, vegetables)	Yang et al. (2024)
Fungal BCAs	*Trichoderma harzianum*, *T. afroharzianum*	Mycoparasitism, CWDEs, antibiosis, ISR	ISR/SAR‐like priming, antioxidant enzymes	*Fusarium*, *Sclerotinia*, *Botrytis*	Harman (2024)
	*Penicillium* spp.	Antibiosis, niche competition, nutrient mobilization	Improved vigor, indirect resistance	Root pathogens (horticultural crops)	Jahan et al. (2024)
Endophytes	Endophytic bacteria and fungi	Internal niche occupation, antimicrobials, hormone modulation	Systemic priming, abiotic stress tolerance	Vascular & foliar pathogens	Narayanan and Glick (2022)
Mycorrhizae (AMF)	*Funneliformis*, *Rhizophagus*	Nutrient competition, immune priming, microbiome modulation	ISR‐like responses, SA–JA balance	Soilborne fungi, nematodes (field crops)	Hashem et al. (2025)

### 6.1 Rhizobacteria (Bacillus, Pseudomonas, Streptomyces)

Plant growth‐promoting rhizobacteria (PGPR) constitute one of the most widely researched and commercialized microbial biocontrol agents. PGPR belonging to the genera *Bacillus, Pseudomonas*, and *Streptomyces* establish themselves in the rhizosphere and rhizoplane, wherein their role is to control disease‐causing microorganisms through processes such as antibiosis, competition, lytic enzyme production, and stimulation of systemic resistance in plants (Sun et al. 2024).

The *Bacillus* group is especially appreciated for its capability to synthesize cyclic lipopeptides like surfactin, iturin, and fengycin, as well as extracellular enzymes like chitinases, proteases, and β‐glucanases that break down the cell walls of pathogens. The formation of resistant endospores and biofilms by *Bacillus* makes them more persistent and effective in excluding pathogens from the environment (Saiyam et al. 2024). Some *Bacillus* strains have shown success in controlling soil‐borne fungi, bacteria, and nematodes in various agricultural environments.

Species of *Pseudomonas* are famous for their capability to produce diverse metabolites with antimicrobial activities, such as phenazine, pyrrolnitrin, and 2,4‐diacetylphloroglucinol (2,4‐DAPG), and siderophores with high affinity for iron. Volatile organic compounds and interference molecules in the quorum‐sensing system produced by *Pseudomonas* species are also responsible for the inhibition of pathogenic fungi, bacteria, and oomycetes (Si et al. 2024). The species of *Streptomyces*, members of *Actinomycetes*, produce many antibiotics and volatile substances with significant antagonistic properties for many plant pathogenic organisms. They have enzyme‐degrading capabilities and can trigger plant defense mechanisms, resulting in disease control along with plant growth promotion (Olanrewaju and Babalola 2019).

In summary, the described rhizobacteria serve as biocontrol agents that incorporate plant disease control and growth stimulation into their functions.

### 6.2 Fungal Biocontrol Agents (*Trichoderma, Penicillium*)

The role of fungal biological control agents (FBCA) is key in reducing the effects of soil pathogens on plants via different mechanisms, some of which include direct and indirect ones. Of all the fungi used in biocontrol, *Trichoderma* is one of the best‐studied groups, having been used extensively for its high competitiveness in colonization of the rhizosphere and rhizoplane (Ayaz et al. 2023). *Trichoderma* spp. engages in mycoparasitism through recognition of hyphae of pathogens, wrapping around them, and releasing enzymes for degrading their cell walls like chitinases and glucanases. They also synthesize a wide range of secondary metabolites with antifungal and antibacterial activities and are strong inducers of systemic resistance in plants (Singh et al. 2024c).

Penicillium spp., despite being studied less extensively than *Trichoderma* spp., have been found to show promising potential as biological control agents against fungi and bacteria through antagonistic actions and habitat displacement. Certain strains of Penicillium also assist in the process of phosphorus mobilization and organic degradation, thereby indirectly benefiting plant health (Sun et al. 2007).

Overall, fungal BCAs combine antagonistic action, immune stimulation, and nutrient cycling, providing practical and sustainable solutions to synthetic fungicides.

### 6.3 Endophytic Microorganisms

Endophytic bacteria and fungi live inside the tissue of plants without inducing any symptoms of disease. This phenomenon is becoming increasingly popular because of their contributions to biological control. Endophytes occupy niches inside the root, stem, and leaf regions of the plants, creating a barrier against invading pathogens, along with the production of antifungal compounds against biotrophs and necrotrophs (Fontana et al. 2021). Endophytes can trigger induced systemic resistance responses, influence hormone pathways, and increase host resistance to abiotic stress factors like drought, salt stress, and extreme temperatures (Pandey et al. 2023). In addition, many endophytes have been observed to synthesize phytohormones and hormone analogs that stimulate growth and simultaneously ensure their immune response capabilities (Pandey et al. 2023). This close connection with their host tissues facilitates their continuous defense activities, making them suitable biocontrol agents for sustained use.

### 6.4 Mycorrhizal Associations

Mycorrhizal fungi such as arbuscular mycorrhizal fungi (AMF) enter a mutually beneficial relationship with the majority of terrestrial plants and have both nutritional and protection purposes. Through the growth of their hyphae network within the soil, AMF assist in phosphorous, micronutrients, and water absorption, which ultimately increases plant strength and decreases vulnerability to infection by pathogens (Xiang et al. 2025).

In addition to their nutritional benefits, mycorrhiza is also involved in biocontrol by stimulating the immunity of plants and activating defense response systems such as ISR and SAR. AMF may be used to decrease the infection by soil fungi and nematodes and to manipulate rhizosphere microflora in a manner that promotes growth of beneficial species at the expense of pathogenic ones (Delaeter et al. 2024).

Microbial groups that include rhizobacteria (*Bacillus, Pseudomonas, Streptomyces*), fungicides (*Trichoderma, Penicillium*), endophytes, and mycorrhizal microorganisms provide multiple and complementary ways of inhibiting pathogens and fostering plant health. Via processes like antibiosis, competition, parasitism, immunization, and improved nutrition, such microbes constitute the ecological basis of bio‐control based on smart ecology. The use of individual strains, selected combinations, and microbiological knowledge‐based inoculants could be an effective strategy to advance towards sustainable protection of crops in the future (Chaudhary et al. 2022).

## 7 Environmental and Soil Factors Influencing Biocontrol Efficiency

The efficacy of biocontrol microbes depends highly on the interplay between soil physio‐chemical characteristics and climatic/abiotic stress as well as the impact of agricultural practices on their activity. These act as key determinants governing microbial survival, metabolism, root colonization, and interaction with both the host plant as well as the pathogen to define the efficacy and stability of biocontrol. Environmental drivers play an integral role in biocontrol and are important for the application of sustainable, eco‐smart disease management approaches based on beneficial microorganisms (Iqbal 2026).

### 7.1 Soil Physicochemical Properties

The physicochemical properties of the soil such as pH, texture, structure, water, organic content, nutrient levels, and oxidation‐reduction status form some of the most important factors that influence the microbial population in the rhizosphere environment. Of all these factors, soil pH emerges as one of the key factors influencing microbial diversity and microbial activity due to its effect on enzyme activity, availability of nutrients, and the relationship between beneficial microorganisms and pathogenic organisms (Xiong et al. 2024). Most bacteria and fungi that exert biological control are more active in neutral soils; deviations from neutrality inhibit their activity.

The properties of soil structure and texture control oxygen diffusion, water holding capacity, and porosity, which affect the respiration, movement, and colonization of microbes in the soil. Fine‐textured soils that lack aeration hinder aerobic biocontrol microorganisms, while coarse‐textured soils with less organic material and moisture prevent microbes from persisting in the soil (Arunrat et al. 2025).

#### 7.1.1 Soil Organic Matter (SOM)

Soil organic matter (SOM) and nutrient availability supply the carbon source and energy required by microorganisms during their metabolic activities. Increased concentrations of SOM lead to increased microorganism biomass, increased diversity, and enhanced biological activity such as production of antimicrobial substances and enzymes (Whalen et al. 2024). Nonetheless, nutrient imbalance in favor of high mineral nitrogen content results in development of rapid copiotrophic microorganisms, which may not be able to suppress plant pathogens. This implies that soil physiochemical optimization must come first for biocontrol agent functionality.

### 7.2 Climate and Abiotic Stress

Climatic factors such as temperature variations, drought, salinity, flooding, and unpredictable precipitation have significant impacts on microbial activity within soils and effectiveness of biological control. Furthermore, plants activate multifaceted physiological and molecular defense networks to cope with adverse abiotic stressors (Praveen et al. 2023a). Temperature influences microbial growth, enzyme reactions, and synthesis of secondary metabolites, whereby both heat and cold stresses can inhibit microbial activities but promote survival of stress‐resistant pathogens (Qu et al. 2023).

Drought and water stress limit soil moisture required for the viability of microbes, transport of metabolic products, and interaction between roots and microbes, resulting in lower efficiency of biocontrol action in dry and semi‐dry environments (Zhakypbek et al. 2026). On the other hand, waterlogged soils experience hypoxia/anoxia that negatively impacts aerobic biocontrol organisms and microbial population composition, possibly supporting anaerobic microorganisms with poor biocontrol ability (Pawar et al. 2025).

#### 7.2.1 Salinity, Sodicity, and Heavy Metal Contamination

Salinity, sodicity, and heavy metal contamination create severe osmotic, ionic stress for both plant and microbe cells. Recent studies highlight that beneficial microorganisms play a pivotal role in mitigating metal‐induced phytotoxicity and enhancing crop environmental resilience (Tiwari et al. 2026; Singh et al. 2026). Although salinity and heavy metals adversely affect native microbes, salt‐ and metal‐tolerant plant growth‐promoting rhizobacteria (PGPR) and fungi can alleviate these toxic impacts by facilitating nutrient acquisition and regulating antioxidant defense pathways (Egamberdieva et al. 2019). In addition to microbial inoculants, advanced material interventions such as engineered nanoparticles have emerged as effective tools in ameliorating heavy metal phytotoxicity and bolstering crop health under harsh soil conditions (Praveen et al. 2023b). Combining these nanotechnological tools with microbial applications offers synergistic potential for sustainable agriculture. These insights highlight the significance of choosing adaptive microorganisms in combating climatic adversities.

### 7.3 Agricultural Management Practices

Agricultural activities have a significant impact on soil conditions, which affect microbial biocontrol efficacy. The frequency of tillage affects soil structure and microbial habitat; high tillage levels cause destruction of fungal hyphal networks and microbial habitat, while low tillage or conservation tillage enhances microbial diversity, stability, and disease suppression in soil (Xing et al. 2025).

Crop rotation and crop diversification change the biochemical composition of root exudates, interrupting the life cycle of pathogens, and promoting microbial communities that inhibit the growth of pathogens (Sun et al. 2024). Likewise, the application rate of fertilizer impacts microbial communities; excessive inorganic fertilizers reduce the richness of microbes, whereas the addition of organic fertilizers like compost increases antagonism (Liu et al. 2025).

In irrigation management, both the levels of moisture and oxygen are important parameters that determine microbial survival and mobility. Irrigation may cause dehydration to the microbial population or cause anaerobic conditions that are not favorable for many biocontrol agents. Lastly, pesticides may have non‐target effects by inhibiting beneficial microbes, hence reducing biocontrol efficiency, especially when using broad‐spectrum fungicides/bactericides multiple times (Kumar et al. 2024).

Combining soil health‐based approaches like reduced tillage, crop rotation, organic matter input, efficient water management, and lower usage of chemicals provides an environment conducive to beneficial microorganisms and increases the effectiveness of biological control methods.

Environmental and soil conditions play a pivotal role in determining the efficacy of biological control agents by influencing the survival, expression, and interactions of microorganisms in the rhizosphere. Factors related to soil chemical and physical attributes, environmental stressors, and agricultural practices will ultimately dictate the ability of these microbes to successfully establish themselves, sustain their presence, and inhibit the activity of pathogens. Understanding how such determinants influence the effectiveness of biological control measures and incorporating them into microbial biocontrol systems development is crucial for achieving sustainable agricultural practices (Iqbal 2026).

## 8 Omics and Artificial Intelligence in Decoding Microbe–Plant Interactions

The rapid development of high‐throughput omics techniques combined with artificial intelligence and machine learning has evolutionized the scientific understanding of plant–microbe interactions. The tools allow for the profiling of microbes in terms of community structure, their functional potential, and activities, as well as revealing molecular and metabolic mechanisms responsible for healthy plant development and protection from diseases. With the help of multi‐omics data and predictive models based on AI, scientists can shift from analysis to the precision design of microbiomes for eco‐smart biocontrol and sustainable agriculture (Sharma et al. 2023; Kimotho and Maina 2024). Schematic representation of an integrated pipeline including multi‐omics approaches and artificial intelligence for eco‐smart biocontrol development, see Table 2. This framework connects microbial community profiling, functional characterization, and predictive modeling with help for making decisions about formulations and in the field.

**Table 2:** Multi‐omics and AI‐driven pipeline for discovery, validation, and deployment of microbial biocontrol agents.

Pipeline stage	Omics/AI layer	Key data generated	Biological insight gained	Application to biocontrol	References
Microbiome profiling	Metagenomics	Taxonomic composition, functional gene repertoire, MAGs	Identification of keystone taxa and biocontrol gene clusters	Selection of candidate BCAs and consortia	Bai et al. (2022)
Functional activity mapping	Metatranscriptomics	Actively expressed microbial and plant genes	Context‐dependent activation of antagonism, ISR, stress pathways	Trait validation under disease/stress conditions	Chialva et al. (2022)
Effector verification	Proteomics	Enzymes, transporters, antimicrobial proteins	Confirmation of expressed biocontrol machinery	Linking genes to real antagonistic function	Pan et al. (2024)
Chemical signaling analysis	Metabolomics	Root exudates, VOCs, antibiotics, phytohormones	Identification of signaling and defense‐eliciting metabolites	ISR priming, pathogen suppression markers	Sibanyoni et al. (2025)
Cross‐layer integration	Systems biology	Interaction networks, metabolic pathways	Discovery of regulatory hubs and trait synergies	Rational design of microbial consortia	Bai et al. (2022)
Pattern Recognition and prediction	Machine learning (ML)	Feature importance, trait–phenotype correlations	Prediction of strain performance and robustness	High‐throughput screening of BCAs	Sadeghi et al. (2022)
Model interpretability	Explainable AI (xAI)	Decision rules, feature attribution	Biological interpretability of AI predictions	Trustworthy strain selection	Ibrahim and Shafiq (2023)
Translational validation	Field phenomics + metadata	Yield, disease severity, climate–soil data	Context‐specific efficacy assessment	Site‐adapted biocontrol deployment	Ahmed et al. (2023)
Product development	AI‐guided formulation design	Stability, survival, compatibility metrics	Prediction of shelf‐life and delivery success	Commercially reliable formulations	Sharma et al. (2023)
Decision support	AI‐assisted agronomic tools	Risk maps, recommendation systems	Precision application guidance	Farmer‐ready eco‐smart biocontrol	Asolo et al. (2024)

### 8.1 Metagenomics, Metatranscriptomics, and Metagenome‐Assembled Genomes (MAGs)

Metagenomics enables culture‐independent assessment of entire microbial communities through shotgun sequencing, providing deep insight into taxonomic profiles and functional gene repertoires (Fadiji and Babalola 2020). The reconstruction of Metagenome‐Assembled Genomes (MAGs) has proven critical for linking specific metabolic capabilities to uncultivated rhizosphere microbiota (Ettinger et al. 2021). Concurrently, metatranscriptomics sheds light on active gene expression, capturing temporal transcriptional dynamics in both microbes and host plants during root colonization and pathogen challenge (Yadav et al. 2023). Moreover, cutting‐edge single‐cell transcriptomics has emerged as a powerful new frontier in plant biotechnology research, offering unprecedented cellular‐resolution insights into transcriptional responses during plant–microbe interactions and stress adaptation (Singh et al. 2024b). Combining metagenomics with metatranscriptomics allows us to understand the difference between genetic capacity and gene function, which is necessary to identify key microbial functions in suppressing pathogens.

### 8.2 Advanced Proteomics and Imaging Metabolomics

Proteomics facilitates the detection and quantification of functional proteins associated with microbial metabolic processes, transport systems, and host immunity modulation (Manzoni et al. 2018).

In parallel, mass spectrometry‐based imaging metabolomics allows high‐resolution spatial mapping of low‐molecular‐weight root exudates including organic acids, amino acids, flavonoids, phenolics, and microbial secondary metabolites. These studies reveal how specific metabolite profiles actively shape disease‐suppressive rhizospheres and prime ISR. Combining metabolomics with transcriptomics and proteomics has been especially effective in demonstrating the functional role of the metabolites implicated in ISR, nutrient uptake, and stress alleviation (Raza et al. 2024; Lv and Fan 2026).

### 8.3 Machine Learning, Deep Learning, and Explainable AI (xAI)

The explosion of multi‐omics data has driven the implementation of advanced machine learning algorithms, including Random Forests, Gradient Boosting Machines, Support Vector Machines, and Deep Neural Networks. These architectures process high‐dimensional datasets to predict microbial functional traits, identify reliable biomarkers of biocontrol success, and forecast strain performance across variable environments (Lin et al. 2025). Furthermore, the integration of Explainable AI (xAI) frameworks demystifies model outputs, allowing researchers to decode the complex nonlinear relationships between microbial community features and plant health outcomes, ultimately enabling data‐informed precision microbiome engineering (Crandall et al. 2020).

This integrative, translational process involving multiple omics discovery, AI‐based microbial screening, formulation, and field trials for eco‐smart biocontrol is highlighted in Figure 3.

**Figure 3:**
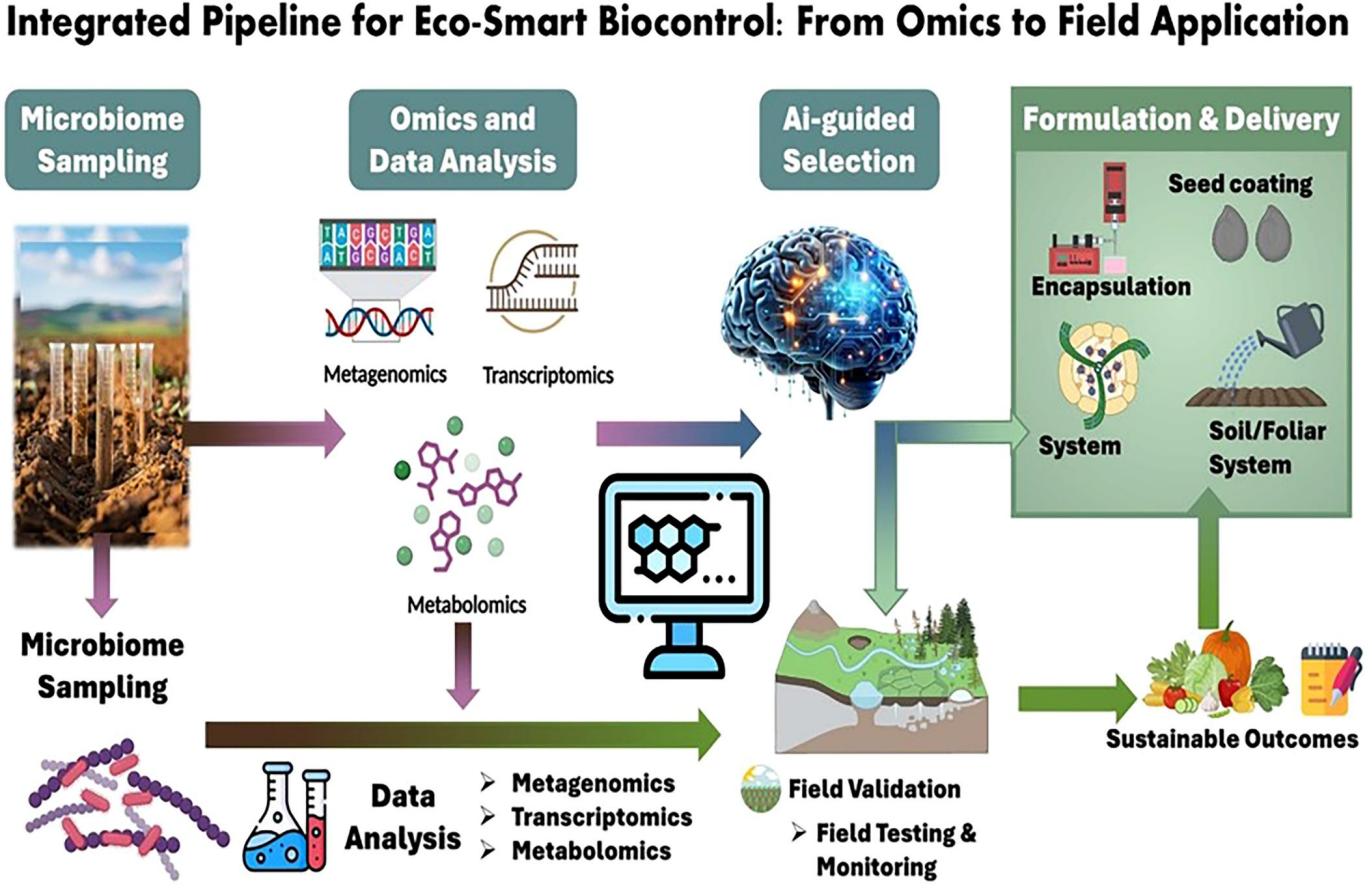
Integrated pipeline for eco‐smart biocontrol: from omics‐driven discovery to field application. Schematic overview of a translational framework for developing eco‐smart biocontrol strategies. The pipeline begins with microbiome sampling from plant–soil interfaces, followed by multi‐omics analyses (metagenomics, transcriptomics, and metabolomics) to resolve community structure, functional potential, and active biochemical interactions. Artificial intelligence and machine‐learning approaches are then employed to identify and prioritize robust microbial strains or consortia. Selected candidates are optimized through formulation and delivery strategies, including seed coating, encapsulation, and soil or foliar application. Field validation and performance monitoring ultimately link laboratory discovery with sustainable agricultural outcomes, such as reduced chemical inputs, enhanced crop productivity, and improved agroecosystem health.

## 9 Formulation and Delivery of Microbial Biocontrol Agents

The efficacy of eco‐biocontrol is not only dependent on the choice of the right strains of bacteria but also on the way in which such strains are formulated and applied to the plant–soil interface without compromising their biological activity and ability to function as beneficial microorganisms under different environmental conditions. Poor formulation has been known as one of the main reasons why microbial BCAs do not show consistent efficacy in the field, mainly due to exposure to harsh climatic factors including desiccation, ultraviolet radiation, extreme temperatures, osmotic shock, and competition from native microbiota (Haq et al. 2024).

### 9.1 Carrier Materials and Encapsulation

#### 9.1.1 Carrier‐Based Formulations

The carrier materials act as a matrix for the delivery of the microorganisms, which (i) keep them alive until their use, (ii) make them easier to handle and apply, and (iii) help them establish themselves once delivered. Examples of common solid carrier materials are talc or clay powder, peat, composted plant materials, lignite or charcoal materials, and agricultural residues (Kumar et al. 2021). Modern studies have concentrated on engineering carriers with additional functionalities like biochar‐containing matrices, biopolymer blends, and nanocellulose‐based preparations. Functional carriers improve moisture content, survival in drought or salinity conditions, and rhizosphere colonization, which are all strongly related to increased field efficacy (Osman et al. 2022).

#### 9.1.2 Liquid Formulations

A significant advantage of liquid formulations is that they are easier to dose, easily compatible with fertigation and drip irrigation, and are easy to spread on a large scale. But for keeping the liquid system stable, strict control over pH, osmolarity, oxygen content, and possible contamination needs to be exercised. Glycerol and other solutes can be used if they do not affect the microorganisms' growth and their biocontrol properties (Allouzi et al. 2022).

#### 9.1.3 Encapsulation as a Protection‐And‐Release Strategy

Encapsulation technology is becoming a popular method of safeguarding microorganisms or spores against external conditions and providing their controlled release in the rhizosphere. Micro‐ and nano‐encapsulation utilizing biopolymers, especially alginate and chitosan, is seen by many as a viable technology for increasing the stability and efficacy of encapsulated versus naked cells (Saberi Riseh et al. 2021).

Encapsulation approaches involve bead‐like structures of alginate and chitosan, microcapsules containing filler substances (such as nanocellulose), encapsulates that are spray or freeze dried, and seed coating habitats. Experimental work shows an improvement in terms of enhanced thermal stability and biological activity in the case of fungal spores when they are encapsulated in alginate‐chitosan systems (Zabot et al. 2022). *Bacillus* spores have been similarly encapsulated to enable protection against stress conditions (Rehman et al. 2026).

#### 9.1.4 Seed Coatings as Targeted Delivery Platforms

Seed coatings are being considered more as a precision approach as they provide the opportunity for BCAs to be present in the very first soil‐root contact area, reduce any possible inoculum loss, and facilitate a microhabitat environment that allows for protection during storage and establishment. Newer approaches in seed coatings involve the use of polymers, films, and sporopollenin capsules that facilitate the survival of microbes without interfering with germination (Rocha et al. 2019).

### 9.2 Shelf‐Life and Stability

Achieving high viability and functionality throughout storage, transportation, and farm management is still one of the most challenging tasks in BCA commercialization. Losses during shelf life are caused by moisture, oxidation, temperature, and formulation issues leading to damage to cell or spore integrity and inhibiting beneficial biological control characteristics (Fenibo and Matambo 2025).

#### 9.2.1 Drying Technologies and Solid Preparations

Formulations in solid form usually exhibit enhanced shelf‐life characteristics and logistical benefits where viability is retained throughout production. Freeze‐drying (lyophilization) and spray‐drying are common techniques used in the manufacture of stabilized biological products, but there exists a strong dependency on strain type and the need for protective agents to avoid injury to membranes and proteins caused by dehydration and rehydration processes (Singh et al. 2023). With respect to *Trichoderma* formulations, researchers recognize that drying conditions, including inlet temperature and excipient properties play a critical role in maintaining conidial viability after storage (Martinez et al. 2023).

#### 9.2.2 Stabilizers and Protective Excipients

Beyond counting viable cells (CFUs or spores), the functional traits such as antagonism, enzyme formation, and induction of host defenses have to be considered. According to the latest reports, monitoring of viability by itself is not enough, since the functional ability can deteriorate despite the presence of viable cells (Prazdnova et al. 2025).

### 9.3 Seed, Soil, and Foliar Applications

#### 9.3.1 Seed Application (Coating, Pelleting, Priming)

Seed‐mediated inoculation is inexpensive and specific in its delivery approach, aiding in early establishment of roots which helps in keeping soil‐borne pathogens under control. Viability of the microorganisms may be affected by conditions such as desiccation, temperature changes, and incompatibility with seed treatment agents. Protection through use of polymers and biofilm formation may be necessary (Singh et al. 2023). Issues that should be considered during this approach include uniformity of the coat, attachment, compatibility with IPM products, and non‐interference with plant growth.

#### 9.3.2 Soil Application (Granules, Drenches, In‐Furrow, Fertigation)

Delivery of soil targets the placement of BCAs right at the site of infection court of the pathogens. The granular forms of formulation and use of carriers help increase persistence through protecting the organisms against environmental stresses as well as placing them closer to the roots. Soils drenches and in‐furrow placements provide fast delivery; however, they may lead to dilution of inoculums, hence the need for a strong formulation.

### 9.4 Foliar Application (Sprays and Phyllosphere Establishment)

Foliar application plays a crucial role in dealing with diseases on the upper surfaces, although it faces certain problems, such as UV radiation, desiccation, rain‐induced runoff, and nutrient starvation. Adjuvants, in such cases, are usually added into formulations to enhance attachment and distribution while keeping microorganisms alive. Current assessments highlight that the choice of adjuvants should not compromise microorganism activities (Arunachalam et al. 2025).

Formulation and application are critical determinants of successful microbial biocontrol agents, serving as the link between lab performance and field applicability. The industry is swiftly moving beyond the concept of using only one strain of microbes in generic formulations to the idea of creating specific formulations through encapsulation, coating seeds, and developing formulations that can survive under stress conditions. This approach to delivery is gaining recognition as key to advancing microbial biocontrol technology (Tadesse Mawcha et al. 2025).

## 10 Case Studies in Eco‐Smart Biocontrol

Case studies validate eco‐friendly biocontrol because they illustrate the effectiveness of beneficial microorganisms under practical agricultural settings rather than artificial laboratory and greenhouse environments. Microbial applications, whether to cereal, legume, or horticultural crops, have been observed to perform satisfactorily in controlling plant diseases and boosting plant health in an economically viable manner through proper formulation and implementation in agricultural production systems.

### 10.1 Cereal Crops

Cereal crops like wheat, rice, and maize are essential for food security on a global scale, yet they are very vulnerable to soil and leaf infections, which limit their yields. Several studies conducted in the field have shown that the application of beneficial microbial inoculants reduces disease incidence and leads to healthier plants (Pawar et al. 2025).

In wheat, the use of AM fungi along with *Trichoderma* spp. has been proven to decrease the incidence of stem and root diseases, increase the activity of antioxidant enzymes like peroxidase and polyphenol oxidase, as well as increase plant biomass and grain production in field conditions (Guzmán‐Guzmán et al. 2023). This is due to enhanced nutrient absorption, immune priming, and pathogen suppression in the rhizosphere.

For example, rice infected with *Rhizoctonia solani* and exposed to plant growth‐promoting rhizobacteria (PGPRs) from the rhizosphere has been reported to exhibit a decline in sheath blight disease accompanied by increased vigor of seedlings, root growth, and dry matter production (Sun et al. 2024). Mixtures of several strains of PGPRs have been especially efficacious.

Cereal‐based case studies collectively demonstrate that microbial inoculants can concurrently improve disease resistance and agronomic performance, providing viable alternatives or supplements to chemical fungicides.

### 10.2 Legume Crops

Legumes are important crops in sustainable farming systems owing to their nitrogen‐fixing capability, but they may be constrained in terms of production by soil pathogens and plant parasitic nematodes. The use of eco‐friendly biocontrol methods in legumes often takes advantage of the interaction between microbial disease control and enhanced symbiosis (Vanlauwe et al. 2019).

Studies conducted in the field on chickpea, pigeon pea, and soybeans have indicated that *Trichoderma* spp. decrease root rot and Fusarium wilt infection rates and promote nodulation, nitrogen fixation, and growth of plants (Chohan et al. 2024). Such activity has been attributed to mycoparasitism, antibiosis, and systemic resistance.

Biocontrol methods have been effective against nematodes in legumes as well. In the case of soybeans and pulses, there is evidence that fungal biocontrol using soil applications of *Purpureocillium lilacinum* (*Paecilomyces lilacinus*) and *Metarhizium anisopliae* has resulted in the reduction of root‐knot nematode population levels and their impact on crop yield (Khan and Tanaka 2023). With PGPR, results become even more favorable.

These legume‐centric case studies highlight the dual benefits of microbial biocontrol in enhancing nitrogen efficiency while safeguarding crops from biotic stress, an integral aspect of eco‐smart intensification.

### 10.3 Horticultural Crops

Horticulture crops tend to be very sensitive to biocontrol agents because of intensive agronomic inputs and high crop management intensity and susceptibility to pest infestations. There have been some significant case studies in the past that highlight the importance of eco‐smart biocontrol in these crops.

In tomatoes, root application of microbial communities comprising *Trichoderma afroharzianum* along with the AMF *Funneliformis mosseae* resulted in increased marketable yield and decreased damage caused by the pest *Tuta absoluta* in commercially grown tomatoes (Almuslimawi et al. 2024). This was attributed to improved nutrition and defense mechanisms, along with resistance to biotic stresses, without affecting the arthropod community.

In fruits, biological control using bacteria like *Pantoea agglomerans* has been effective against *Erwinia amylovora* infections, known as fire blight, in apple and pear plantations. There are many cases where the biocontrol agent has been proven to inhibit the pathogens by competing for niches and showing antibiosis activity, with effectiveness comparable to chemicals (Dagher et al. 2020).

Horticultural case studies consistently demonstrate that microbial biocontrol agents can be incorporated into integrated pest management (IPM) strategies, ensuring compatibility with natural enemies and diminishing reliance on pesticides without sacrificing yield or quality.

In cereals, legumes, and vegetables, examples of ecological biocontrol applications show how beneficial organisms can: (i) minimize the occurrence and intensity of diseases by means of antagonism, competition, parasitism, and induced resistance, (ii) increase yield and crop quality during field cultivation and industrial production, and (iii) be easily incorporated into IPM schemes and sustainable agriculture practices (Pandit et al. 2022). These examples show how knowledge obtained through understanding mechanistic aspects of plant–microbe interactions can be applied in an efficient and sustainable manner. It is also important to note that consistent success in this endeavor requires appropriate selection of microbial strains, formulation methods, and inoculation techniques for crops, soils, and agricultural systems, respectively, thus emphasizing the fundamental tenets of eco‐smart biocontrol. Examples of eco‐smart biocontrol in different crop production systems are provided in Figure 4.

**Figure 4:**
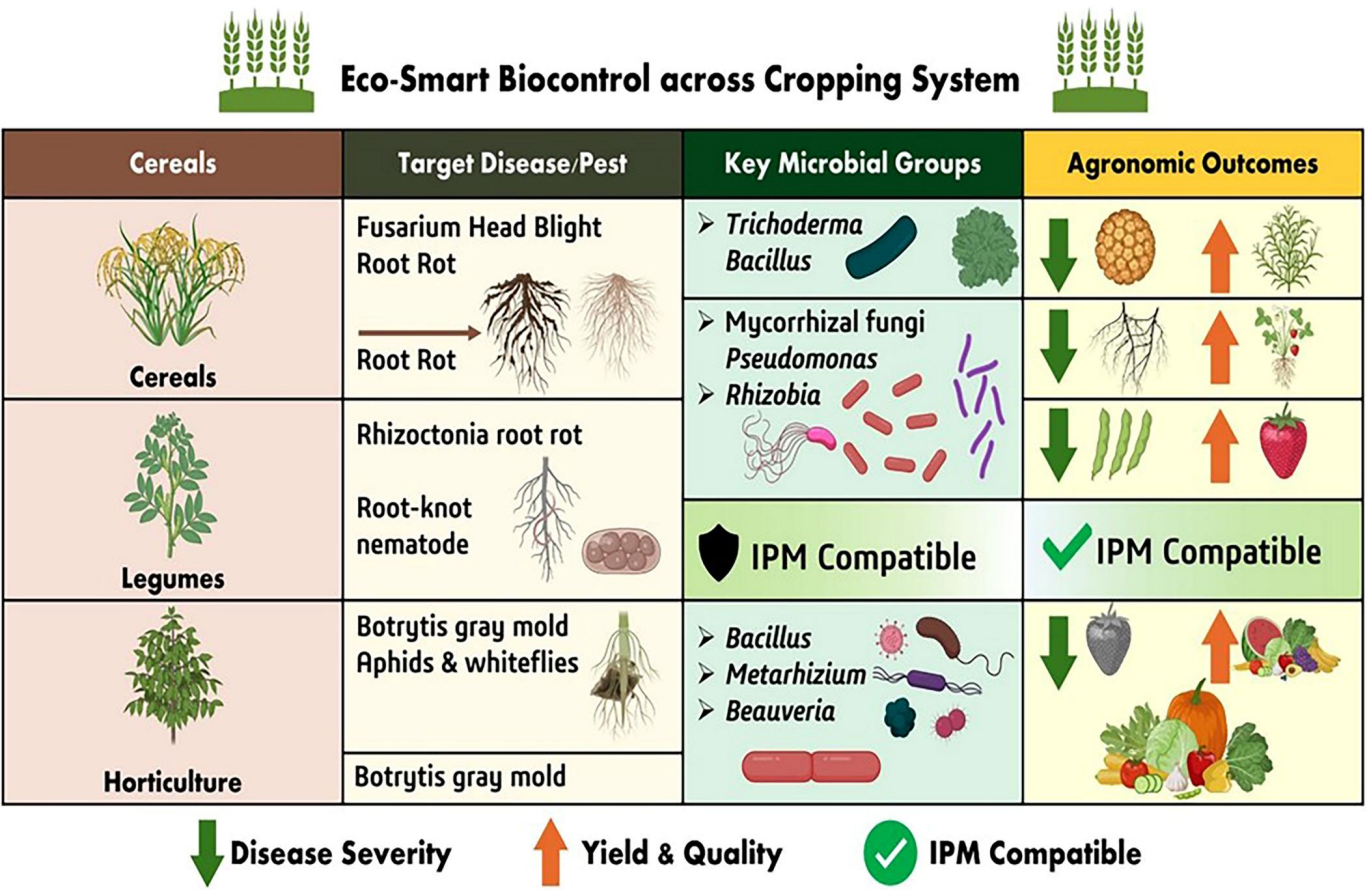
Translation of eco‐smart biocontrol across major cropping systems. Summary of representative case studies demonstrating the application of eco‐smart biocontrol strategies in cereals, legumes, and horticultural crops. The figure highlights key target diseases or pests, dominant beneficial microbial groups involved (including *Trichoderma*, *Bacillus*, *Pseudomonas*, mycorrhizal fungi, and entomopathogenic fungi) and associated agronomic outcomes. Across cropping systems, microbial inoculants consistently reduce disease severity and pest pressure, enhance yield and crop quality, and remain compatible with integrated pest management (IPM) practices, underscoring their broad applicability and practical relevance in sustainable agriculture.

## 11 Challenges and Limitations

However, even with great success, the widespread use of microbial biocontrol is hindered by a number of issues relating to environmental, technological, and regulatory challenges. Important among these issues are unpredictability in field effectiveness, establishment, and competitiveness of the introduced microbes. Overcoming such obstacles is imperative for the effective use of eco‐friendly biocontrol practices in agricultural production (Iqbal 2026). Environmental, technological, and regulatory issues that constrain microbial biocontrol practices and the eco‐friendly solutions to overcome these challenges have been outlined in Table 3.

**Table 3:** Key constraints limiting microbial biocontrol performance and corresponding mitigation strategies within eco‐smart frameworks.

Constraint category	Specific limitation	Impact on biocontrol performance	Mitigation/eco‐smart strategy	References
Soil physicochemical factors	Extreme pH, low SOM	Reduced microbial survival, suppressed metabolite production	Soil amendments, strain selection for pH tolerance	Delgado‐Baquerizo et al. (2022)
Climate and abiotic stress	Heat, drought, UV exposure	Rapid decline in cell/spore viability	Stress‐tolerant strains, encapsulation, and seed delivery	Sharma et al. (2023)
Microbial competition	Dominance of native microbiota	Poor establishment of introduced BCAs	Consortia design, biofilm‐supportive carriers	Ahmed et al. (2023)
Formulation limitations	Short shelf‐life, trait loss	Reduced field efficacy despite high CFU	Encapsulation, stabilizers, dry formulations	Balla et al. (2022)
Delivery constraints	Poor root/leaf colonization	Inconsistent disease suppression	Seed coating, in‐furrow delivery, and adjuvants	Elnahal et al. (2022)
Regulatory barriers	Complex, non‐harmonized approval	Delayed commercialization	Science‐based, harmonized risk frameworks	Bonaterra et al. (2022)
Adoption barriers	Farmer risk perception	Low uptake despite efficacy	Extension, IPM integration, field demos	Sharma et al. (2023)

### 11.1 Field Inconsistency

Field inconsistency is still a critical impediment for the utilization of microbial biocontrol. Although several biocontrol organisms have shown excellent antagonistic effects in the laboratory or greenhouse settings, their effectiveness may become compromised or variable when applied in the field, which presents changing environmental conditions of abiotic and biotic nature (Rezaee Danesh et al. 2025). Different types of soil, water supply, extreme temperatures, UV light exposure, and seasonal changes could have significant impacts on the performance of microorganisms.

Environmental stress factors like heat and ultraviolet radiation can quickly diminish microbial viability after application, especially for bacteria that do not produce spores or have poor formulation protection (Pazos‐Rojas et al. 2023). Moreover, genetic diversity among plants, crop cycles, and pathogen populations can cause site‐specific effects, where an identical microbial treatment may be effective in one region or season but ineffective elsewhere (Fadiji et al. 2025).

This variability makes growers less confident and makes it harder to include them in standard disease management programs. This shows how important it is to have formulation strategies, strain selection, and deployment protocols that take the environment into account.

### 11.2 Microbial Survival, Establishment, and Competition

Another important problem that needs to be considered is the low survivability and competitiveness of introduced biological control agents in the existing microbiome of soil and plants. The local microflora is usually adapted to the environment and can compete with the introduced microorganisms for nutrition and space, thus preventing the spread and longevity of biocontrol agents (Fadiji et al. 2025).

A sensitivity to abiotic stresses is yet another limitation that affects the survival of the microorganisms in the wild. The changes in the amount of moisture content in the soil, pH levels, temperatures, and salinity may hinder the multiplication of the microbes and their metabolism, especially if these organisms have not been specifically developed for stress tolerance (Ullah et al. 2025). Moreover, the products derived from microbes do not possess long shelf lives.

As solutions to these problems, the focus on microbial consortia consisting of strains with complementary abilities has gained momentum. Despite the potential benefits offered by consortia, such systems present new problems, including strain compatibility, stability, formulation, and regulatory hurdles (Belda 2026).

### 11.3 Regulatory, Biosafety, and Adoption Barriers

Regulatory and biosafety frameworks pose substantial hurdles to commercialization for microbial control systems. Although microbial pest control agents are generally perceived as posing lower environmental risks than synthetic chemicals, their registration involves rigorous evaluation criteria (Baud et al. 2026). A comparative analysis of major global regulatory pathways reveals distinct operational paradigms:

United States (Environmental Protection Agency—EPA): Governed primarily under the Federal Insecticide, Fungicide, and Rodenticide Act (FIFRA). The EPA utilizes a tiered testing framework focusing on acute mammalian toxicity, pathogenicity assessments, and environmental fate, supported by expedited review programs for microbial and biochemical pesticides.

European Union (European Commission—Regulation [EC] No 1107/2009): Enforces highly stringent data requirements regarding active substance approval, environmental persistence, and non‐target organism safety. The rigorous data burden and hazard‐based cut‐off criteria frequently result in longer market entry timelines for microbial biopesticides in Europe (Baud et al. 2026; Fernández Ríos et al. 2025).

FAO/Codex Alimentarius: Provides international harmonization guidelines, maximum residue limit (MRL) exemptions for biological agents, and standardized safety evaluations to assist developing nations in establishing science‐based regulatory protocols (Fernández Ríos et al. 2025).

Beyond regulation, widespread adoption is constrained by grower perceptions, limited local extension services, and a lack of region‐specific field validations (Pawar et al. 2025). Overcoming these barriers requires aligned global regulatory standards, adaptive strain selection, and structured educational outreach (Pawar et al. 2025; Zhou et al. 2024).

## 12 Conclusion and Future Perspectives

Eco‐smart biocontrol provides a new paradigm for sustainable protection against crop pests by exploiting the biological functionality of microorganisms associated with plant roots. Eco‐smart biocontrol uses microorganisms in the rhizosphere to promote plant growth, control plant diseases, and minimize the use of artificial pesticides. This review examines the rhizosphere as a very active and interacting environment where bacteria, fungi, actinomycetes, archaea, and protists participate in several networks involving nutrient sharing, biochemical interactions, and succession of their populations over time.

Insights from the mechanistic point of view have shown that microbial biocontrol involves multiple strategies, both synergizing, such as antibiosis and secondary metabolites, quorum sensing disruption, biofilm formation on the roots for colonization, and regulating plant phytohormones and plant immunity signaling pathways. Indeed, the development of advanced omics, systems biology, and artificial intelligence technologies has further revolutionized the field, facilitating detailed analysis of plant–microbe interaction networks, discovery of key microbes and their associated traits, and selection of microbial communities. Case studies from cereals, legumes, and horticultural plants demonstrate that such insights can indeed lead to improvements in plant productivity.

Despite all these achievements, there are certain limitations that still restrict its wide application. The first one is the lack of consistent performance of these agents in the field. Second, it is difficult for foreign microorganisms to survive in the field and compete with native organisms. Third, there are regulatory issues and biosafety concerns.

Moving forward, the way forward for sustainable biocontrol through eco‐smart practices involves shifting from trial‐and‐error approaches based on single strains to predictive engineering of microbiomes. This would involve leveraging multi‐omics data along with artificial intelligence models to rationally design microbial communities that can thrive under specific cropping systems, soils, and climates with improved stability and resilience. Increased focus on large‐scale field trials across various agroecosystems will be vital to account for uncertainties related to changing climatic patterns. The emergence of delivery technologies, including smart encapsulation, improved seed coating, and stress‐tolerant formulations, is anticipated to help achieve greater efficacy of microbial inoculants. Concurrently, the evolution of science‐based regulatory guidelines that facilitate global adoption will be crucial for successful implementation.

To conclude, eco‐smart microbial biocontrol represents a holistic approach towards sustainable agricultural practices that is environmentally friendly and future‐oriented. An integration of ecological principles, a knowledge base about how microbes work, a multi‐omics knowledge base, AI‐enabled predictive modeling, and field‐based validations will lead to the development of the next generation of biocontrol measures that are robust, scalable, and adaptive for different farming practices.

## Author Contributions

**Shilpy Singh:** conceptualization, investigation, writing – original draft, methodology, supervision, data curation. **Varun Kumar Sharma:** investigation, methodology, validation, data curationv supervision. **Dharmsheel Shrivastav:** formal analysis, data curation, supervision, writing – review and editing. **Jayant M. Kushwaha:** investigation, formal analysis, supervision, writing – review and editing. **Manoj Kumar Mishra:** visualization, validation, writing – review and editing. **Mirza Masroor Ali Beg:** conceptualization, investigation, writing – review and editing, supervision, formal analysis.

## Funding

The authors have nothing to report.

## Ethics Statement

The authors have nothing to report.

## Conflicts of Interest

The authors declare no conflicts of interest.

## AI Usage Statement

Artificial intelligence (AI) tools were used to assist language editing and grammar correction. No AI tools were used to generate original scientific results or data interpretations.

## Data Availability

Data sharing is not applicable to this article as no data sets were generated or analyzed during the current study.
